# Patients’ reasons for declining a primary care trial online therapy: a mixed-methods study

**DOI:** 10.3399/BJGPO.2024.0272

**Published:** 2025-07-30

**Authors:** Fiona Fox, Debbie Tallon, Roz Shafran, Paul Lanham, Christopher Williams, Berry Jude, Nicola Wiles, David Kessler, Katrina Turner

**Affiliations:** 1 NIHR ARC West, Bristol, UK; 2 Population Health Sciences, Bristol Medical School, University of Bristol, Bristol, UK; 3 Centre for Academic Mental Health, Population Health Sciences, Bristol Medical School, University of Bristol, Bristol, UK; 4 Great Ormond Street Institute of Child Health, University College London, London, UK; 5 Public and Patient Involvement Representative, London, UK; 6 Institute of Health and Wellbeing, University of Glasgow, Glasgow, UK; 7 Five Areas Ltd, Titan Enterprise Business Centre, West Dunbartonshire, UK; 8 UCL Division of Psychiatry, Maple House, London, UK

**Keywords:** depression, cognitive behavioural therapy, randomised controlled trials, mental health, primary health care

## Abstract

**Background:**

Integrating therapist-led sessions and cognitive behavioural therapy (CBT) materials within one online platform may be effective for people with depression. A trial evaluating this mode of delivering CBT is being conducted. To maximise future trial recruitment and understand patients’ views of health interventions, it is important to explore reasons for declining to participate.

**Aim:**

To explore patients’ reasons for declining to participate in a trial of integrated online CBT for depression.

**Design & setting:**

A mixed-methods study collecting data from patients via questionnaires and telephone interviews at three UK trial sites.

**Method:**

Individuals completed a short questionnaire about their reasons for not taking part in the trial. Telephone interviews further explored these reasons with a subgroup. Quantitative data were summarised using descriptive statistics. Qualitative interviews were analysed thematically.

**Results:**

Of 1799 patients who responded to an invitation to participate in the trial, 40.3% declined contact. The most common reasons were not wanting: to take part in research (*n* = 387); therapy provided online (*n* = 284); to receive CBT (*n* = 262). Qualitative interviews with 15 ‘decliners’ highlighted that decisions related to perceptions of eligibility, previous experiences of CBT, and uncertainty about receiving CBT online. Personal circumstances, depressive symptoms, or other mental health issues were also barriers to participation.

**Conclusion:**

Reasons given by primary care patients for not taking part in a trial of integrated online CBT suggest that, at the point of recruitment, it is important to discuss the patient’s perceptions of their eligibility and whether they would accept the intervention being evaluated.

## How this fits in

Clinical trials are needed to evaluate new ways of delivering cognitive behavioural therapy (CBT) for depression in primary care. Recruitment to mental health trials in primary care is often challenging, affecting the quality of evidence they provide. Patients may decline to take part in research owing to mistrust about the research process or because they do not want to engage with the treatment being evaluated. Patients’ previous treatment experiences and perceptions of their health may be key reasons for declining to take part in a clinical trial. When referring patients to a trial, practitioners could explore whether patients view themselves as eligible and would accept the intervention being evaluated. When referring patients for online CBT, GPs could explore patients’ trust in the online setting and their experience of and views about CBT.

## Introduction

Cognitive behavioural therapy (CBT) is recommended for varying severities of depression.^1^ In the NHS, CBT is usually delivered via NHS talking therapies but access to this service varies across the UK owing to pressure on services.^2^ Given rising demand, it is important to explore new, accessible ways of providing CBT in primary care that are acceptable, effective, and cost-effective.^3^

Online-based CBT interventions have been recommended by the National Institute for Health and Care Excellence (NICE) since 2009 for the treatment of depression.^4^ These online interventions increase accessibility and availability^5,6^ at lower cost than in-person therapy.^6^ Interventions range from self-guided approaches, with limited therapist input, to hybrid interventions, which include in-person contact with a therapist. Programmes where therapists deliver CBT sessions, via real-time instant messaging, can be as effective as in-person CBT.^7–9^

A multi-centre randomised controlled trial (INTERACT) is evaluating the clinical and cost-effectiveness of integrated online CBT for primary care patients with depression in reducing depressive symptoms and improving quality of life over 12 months, compared with usual GP care. This mode of CBT delivery combines live high-intensity sessions from an accredited therapist delivered by typed instant messaging, with CBT resources (videos, worksheets, and information sheets) via an online platform.^10^

Effective recruitment to trials is crucial to successfully provide robust evidence^11,12^ and to the generalisability of research findings to routine clinical practice. Individuals may decline to take part in mental health trials owing to uncertainty about research, or the intervention being evaluated.^13,14^ Depressive symptoms may adversely affect patients’ ability and motivation to participate in research.^15,16^ To optimise recruitment to the INTERACT trial, and to improve future trial methodology, questionnaire and interview data were collected from individuals who declined to take part, to understand reasons for non-participation. This article presents findings from these data, providing insights into primary care patients’ views of research and integrated online CBT.

## Method

### The INTERACT trial decliner study

Participants were recruited to the INTERACT trial^10^ through GP surgeries in Bristol, London, and York, either via a referral following a GP consultation or by mailout from their GP practice. Inclusion criteria for trial participants were people aged ≥18 years, who scored ≥14 on the Beck Depression Inventory (BDI-II), and who met International Classification of Diseases 10th revision (ICD-10) criteria for depression. Exclusion criteria included substance dependency; specific mental health conditions; recent receipt of CBT; and unwillingness to receive CBT via computer, laptop, tablet, or smartphone.^10^

**Figure 1.**
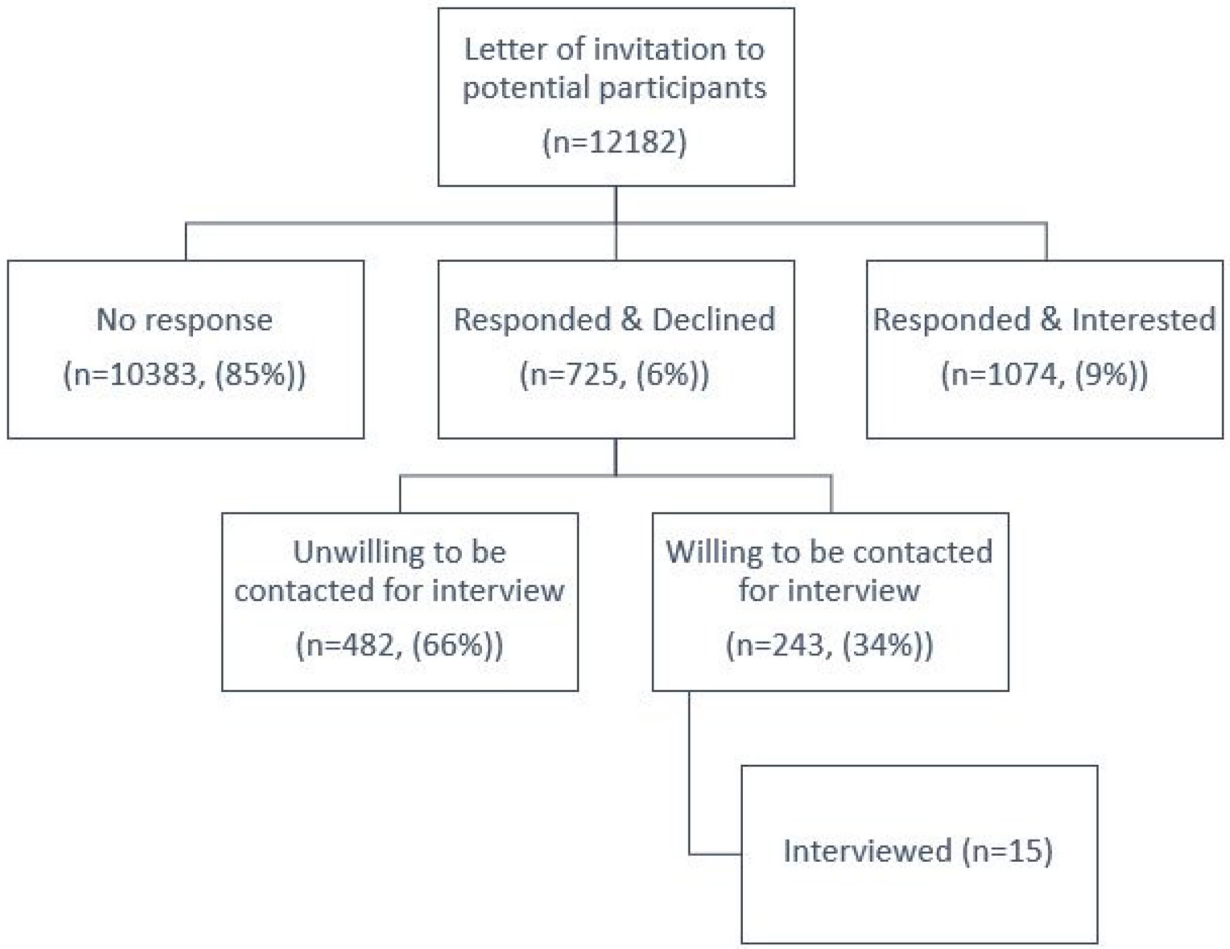
Recruitment flowchart

The data reported in this study are from mailout responders only. These individuals were identified through systematic searches of GP practice records of depression. The mailout included an invitation letter, study information leaflet, reply slip, and a pre-paid envelope. The reply slip asked the recipient to respond if they were willing to be contacted by a researcher to discuss participation in the trial. If they did not want to take part, they were asked to indicate this and complete a short questionnaire about their reasons for declining (see Figure 1: recruitment flowchart).

### Questionnaire: data collection and analysis

The questionnaire was part of the reply slip and asked for the responder’s age, gender, and reason(s) for declining participation, using a series of 11 closed responses (Table 1) and one open ‘other’ category. These items were based on previous research.^17^ Individuals could tick all boxes that applied. Individuals were also asked to indicate whether they would be willing to take part in a short telephone interview to discuss their reasons for declining in more detail. These data were collected throughout the entire trial recruitment period (December 2020–2 April 2023).

**Table 1.** Reasons for declining to be contacted about the INTERACT trial

Reason	*n*	%
I do not want to take part in a research study	387	53.4
I do not want to receive therapy that is provided online	284	39.2
I do not want to receive CBT	262	36.1
I do not feel I would benefit from CBT	246	33.9
I take antidepressants and don’t require any additional treatment	206	28.4
I am not depressed	188	25.9
I have previously received CBT	175	24.1
I do not have access to a computer, tablet, or smartphone	115	15.9
I am currently receiving a talking therapy	107	14.8
I am too busy for CBT and/or research	90	12.4
I am on a waiting list to receive CBT	28	3.9
Other reason	221	30.5

Individuals were able to indicate more than one reason for declining, hence percentages do not add up to 100%. CBT = cognitive behavioural therapy

Analysis of the quantitative data was undertaken using Stata (version 18.0). Descriptive statistics were used to describe the age and gender of potential study participants and reasons for declining to be contacted. The free-text responses explaining ‘other’ reasons for declining were analysed thematically by one researcher (FF) and independently reviewed by a second qualitative researcher (KT).

### Interviews: recruitment and sampling

Interviews were conducted with a sub-sample (*n* = 15) of those who had returned the decliner questionnaire and indicated willingness to take part in a telephone interview (*n* = 243). In order to gain insight into the acceptability of key aspects of the trial and the intervention, the researcher purposively sampled individuals who had given one or more of the following reasons on their questionnaire: I do not want to take part in a research study; I do not want to receive CBT; I do not feel I would benefit from CBT; I do not want to receive therapy that is provided online. Within this, the researcher aimed for maximum variation in terms of participant age, gender, and trial site. Individuals sampled for the interview were contacted to discuss taking part in an interview and, if they were still willing, to arrange a time. The sample provided sufficient information power to meet the aims of the study.^18^

### Semi-structured interviews: data collection and analysis

The interviews were conducted by telephone by an experienced qualitative researcher (FF) between November 2021 and March 2023. Telephone interviews are a methodologically robust alternative to in-person interviews^19,20^ and were appropriate for the relatively brief nature of the discussion.

Verbal consent to participate and to audio-record the interview was secured before the interview. To maintain consistency across interviews, the researcher used a topic guide covering experiences of receiving the invitation letter and information, reasons for declining the invitation, and depression management. The acceptability of communicating with therapists via instant messaging was also discussed. With responders’ consent, all interviews were transcribed verbatim by a professional General Data Protection Regulation (GDPR)-compliant company and then anonymised by FF to remove any identifying information, before analysis.

Interview transcripts were imported into NVivo (version 14) and coded for themes and sub-themes to develop a coding frame. This frame was reviewed and discussed until consensus was reached with a second researcher (KT), who had also coded one-third of the interview transcripts. Following coding, data pertaining to each code was summarised in tables using an approach based on framework analysis.^21^ Comparisons were then made within and across the interviews to identify thematic patterns and deviant cases.

## Results

### Quantitative results

Those who responded to the invitation letter from the GP were older (*n* = 1649, mean age = 48.5 years [standard deviation {SD} = 18.3 years]) than those who did not respond (*n* = 9950; mean age = 40.4 years [SD = 15.6]; t-test: *P*<0.0001). Females were more likely to respond to the invitation to participate than males (responders, *n* with available data = 1659, *n* female = 1119 [67.5%] versus non-responders: *n* = 10 022, *n* female = 6377 [63.6%]; χ^2^ test: *P* = 0.003). Among those who responded, those who agreed to be contacted were younger (*t*-test: *P*<0.0001) than those who declined contact, but there were no differences in gender (χ^2^ test: *P* = 0.56). Among those who declined the invitation, those who were willing to be interviewed about their reasons for declining were younger (*t*-test: *P* = 0.0026) than those who were not, but there were no differences in gender (χ^2^ test: *P* = 0.91). Characteristics of invitees, non-responders and responders are presented in Table 2.

**Table 2.** Comparison of age, gender, and trial site of those who did or did not respond to the invitation to participate

Characteristic	Invited	Non-responders	Responders		
			Agreed to contact	Declined contact	Agreed to take part in telephone interview about reasons for declining
**Mean age, years (SD),^a^** ***n***	41.5 (16.2) *n* = 11 599	40.4 (15.6) *n* = 9950	43.1 (15.9) *n* = 990	56.7 (18.5) *n* = 659	53.7 (17.9) *n* = 226
**Gender: female,^a^** ***n*** **(%)**	7496 (64.2)	6377 (63.6)	665 (66.9)	454 (68.3)	157 (68.6)
**Trial site:** ***n*** **(%)**					
Bristol	4362 (35.8)	3604 (34.7)	462 (43.0)	296 (40.8)	108 (44.4)
London	5080 (41.7)	4495 (43.3)	358 (33.3)	227 (31.3)	79 (32.5)
York	2740 (22.5)	2284 (22.0)	254 (23.6)	202 (27.9)	56 (23.0)

^a^Data on age and gender were not available for all. *n* denotes those with available data. SD = standard deviation

Of the 725 individuals who declined to be contacted by the research team, 709 gave one or more reasons for declining. The most common reason for declining was ‘I do not want to take part in a research study’ (*n* = 387) followed by ‘I do not want to receive therapy that is provided online’ (*n* = 284) (Table 1). Of those who returned a reply slip, *n* = 488 cited one or more of the four reasons for declining that had been selected as criteria for the qualitative interviews. Two-hundred and twelve provided a free-text reason in the ‘other’ reason box and qualitative analysis of these data resulted in the following five themes: Timing not right; Other physical health problems; Other mental health reasons; Do not want CBT; Do not want online integrated CBT (Table 3).

**Table 3.** Other reasons for declining: main themes and sub-themes

**Timing not right:** Lack time; moving house; family problems; other responsibilities.
**Other physical health problems:** Other health conditions more of a priority; health problems affect participation, for example, fatigue or hearing impairment.
**Other mental health reasons:** Do not feel depressed; anxiety more of a problem; mental health improved.
**Do not want CBT:** Dislike CBT; negative previous experiences; have recently had or currently having CBT; currently having different treatment; preference for different treatment.
**Do not want online integrated CBT:** Poor technology skills; prefer in-person therapy; can’t concentrate online; lack privacy at home.

CBT = cognitive behavioural therapy

### Interview findings

Telephone interviews were conducted with 15 people (Table 4). Eight were male and seven were female. The average age was 51 years. Interviews lasted between 10 minutes and 30 minutes (average 18 minutes).

**Table 4.** Characteristics of those interviewed (*n* = 15)

Characteristic	Measure
**Site:** *n* (%)	
Bristol	9 (60.0)
London	5 (33.3)
York	1 (6.7)
**Gender, female:** ***n*** **(%)**	7 (46.7)
**Mean age, years (SD) [range]**	51 (18.6) [20–80]

Analysis of the interview data indicated the main reasons individuals declined to take part in the trial related to their (1) perceptions of their mental health; (2) previous experiences of CBT; and (3) willingness to engage with online CBT. The findings are presented accordingly and quotes are tagged with the interviewee’s number, gender, and age in years.

#### Theme 1: Perception of mental health diagnosis, status, and management

##### I’m not depressed

Some individuals were effectively managing their depression through lifestyle changes or by taking antidepressants. They did not currently view themselves as depressed and therefore thought they would not be eligible to take part in the trial, and that others would benefit more from participation. One interviewee, who no longer felt depressed, explained they were worried they would skew the data:

*‘The more I thought about it the more I thought it wouldn’t quite be the right thing for the research group rather than me … I like to think that you have a good enough study sample to be able to do that without potentially someone maybe whose figures don’t really represent true analysis of how things ought to be.*’ (PT5, male, 64)

Some did not believe they matched the study criteria because they did not consider themselves depressed but had another mental health difficulty:

*‘I wouldn’t say it was depression — see I have never called it depression — anxiety yes maybe and a type of phobia but I would never say it was a depression.’* (PT6, male, 73)

##### Depression is not my main problem

More than half of the participants interviewed (*n* = 8/15) accepted they were depressed but excluded themselves because they felt depression was not their main mental health problem. Instead, they viewed anxiety — including phobias, health anxiety, and panic — or personality disorders, as their main mental health problem:

*‘I suppose if it (the study) had been anxiety based — well I mean health anxiety was my main problem to be fair.’* (PT7, female, 80)

The presence of other mental health issues was important, as it sometimes led to the individual having a negative reaction to the study invitation, which they found hard to cope with. A few interviewees reported that because the study invitation was unexpected, it caused some distress:

*‘It triggered actually because I wasn’t expecting it … As well as depression I have emotional, what they used to call BPD (Borderline Personality Disorder) and I’ve done DBT (Dialectical Behavioural Therapy) … so, it just triggered a bad reaction … It might be worth checking that when people are referred that they have just depression, and they haven’t got anything else that could trigger off a negative response.’* (PT3, female, 55)

##### It’s not the right time

The decision not to participate was commonly linked to the individual’s mental health at the time of receiving the study invitation. This could be because they felt they were managing well and not in need of CBT; they were recovering from depression and were concerned that receiving the intervention may lead to a deterioration in their mental health; or were too fragile to take part:

*‘So, I was probably still in my, I’m still feeling a bit fragile state, rather than feeling a bit more like in recovery.’* (PT1, male, 46)

One participant explained that the study invitation *‘just needs to hit at the right time’* (PT14, female, 42) when an individual is seeking or feeling ready to engage with therapy.

### Theme 2: Suitability for CBT: previous experiences

Some interviewees decided not to participate owing to negative experiences of previous counselling or CBT. Those who had previously found CBT helpful (either group CBT or one-to-one low-intensity CBT) did not feel they needed it again and some emphasised the short-term benefits of CBT:

*‘It was helpful at the time but then COVID hit and it all went a bit skew-whiff* … so *for me personally I’m not sure how much long-term benefit there is*.’ (PT14, female, 42)

One participant found engaging with the written aspects of CBT and the homework particularly problematic:

*‘I don’t really get on with CBT. I’ve tried it a couple of times before so I thought I didn’t really want to waste their (researchers’) time … I started to really struggle because there were always the workbooks and stuff to do. I just really struggle actually sitting down and making time to do them so I don’t get the benefit of it because I’m not doing the work as such*.’ (PT8, male, 30)

### Theme 3: Willingness to engage with online CBT

#### Client–therapist rapport

Participants of all ages expressed their preference for in-person therapy over therapy delivered online. Some felt that leaving the house to attend therapy was an important part of tackling depression. They indicated that in-person sessions would improve the therapist–client connection and rapport, which was crucial to facilitate communication:

*‘The actual contact and actually talking with a person as opposed to going on a screen, to me was very important, slightly old-fashioned way of doing it I understand. I think with something like that when it took me a while to sort of go along with treatment and things like that, that the actual having the personal contact was developing a rapport with the person. I am not saying you can’t do it online or on a screen … I think personal contact is huge because that’s a big step to start talking in my view*.’ (PT4, female, 72)

#### Too much time online

Interviewees’ comments indicated a misperception that the intervention would involve videocall sessions with a therapist. Although the initial session was delivered by videocall, all subsequent sessions were mediated via typing. This suggests they had not fully understood the mode of delivery when deciding to decline. Within the context of the COVID-19 pandemic, some responders felt that they were already spending too much time videocalling:

*‘I was desperate at the time to get away from spending more time on Zoom … So the thought of spending any more time on Zoom was just the opposite of what I wanted*.’ (PT1, male, 46)

This participant elaborated that engaging in online therapy could feel like, *‘more work, as opposed to something that would help me feel better’* (PT1, male, 46).

#### Online format is less accessible and desirable

Age was cited as a factor affecting the acceptability of online CBT, relating to technological skills, or to comfort with conversing online:

*‘Maybe a lot of people — it is relatively a new technology I suppose of doing things online, maybe people of a certain age wouldn’t understand computers.’* (PT6, male, 73)

When the researcher explained that CBT was delivered online via typed communication, some participants raised concerns, related to the speed and accuracy of typing and the challenge of conveying meaning via written messages:

*‘It certainly seems more accessible. For me personally I would prefer to speak to someone over the phone, over a videocall or in person because just trying to convey meaning in text is quite hard sometimes.’* (PT8, male, 30)

Specific barriers, such as dyslexia, were also raised:

*‘As a dyslexic I would really struggle with that (typing) … Like I can get my thoughts generally in a sensible order from my brain to my mouth. But from my brain to my hands, my thoughts just don’t work that way*.’ (PT12, female, 35)

It was acknowledged that online therapy may be more acceptable given recent trends towards online interactions between professionals and patients:

*‘I think we — the nation as a whole, with the recent pandemic … are having to accept moving away from a face-to-face* [in-person] *contact with a medical professional and you can get an awful lot more done with written things … it is less personal but it is more focused.’* (PT5, male, 64)

#### Mistrust of the online setting

Several interviewees thought that privacy could be compromised when using an online platform, which was of particular concern when discussing sensitive issues related to mental health. Some were mistrustful of receiving therapy from someone they could not see and felt that anxiety or ‘*paranoia*’ about this may be common for people with depression. The following quote reflects a misunderstanding that the trial intervention was solely delivered via Zoom videocall but highlights concerns that may be exacerbated if the client and therapist cannot see each other at all:

*‘When you’re sat in a room with somebody you know it’s just you and that one person sat in the room … but when you’re doing it on Zoom, I don’t know what you’ve got in that room … I don’t know if there’s anybody else sat behind the computer that I can’t see … you could have 20 of your mates behind the camera. I know you wouldn’t but — with the way that our minds work, the paranoia and stuff*.’ (PT2, female, 49)

One participant expressed an angry response to the idea of online CBT, commenting:

*‘The government is just trying to make it (therapy) as cheap as they can. They don’t have the staff to help people and they’re just trying to pretend that they are helping people even on a screen. It’s just a cruel joke*.’ (PT14, male, 48)

## Discussion

### Summary

The quantitative data showed the most common reason for declining trial participation was not wanting to take part in research. Analysis of free-text data showed that this was often related to current personal circumstances and health issues. When combined, the reasons concerning CBT (‘I do not want CBT’ and ‘I would not benefit from CBT’) were the most commonly cited reason. The open and flexible nature of the interviews allowed us to explore in more detail participants’ reasons for not wanting CBT. Those who did not find CBT helpful, cited difficulties with the written aspects and homework. Those who had found it useful felt no need to repeat the process. While receiving therapy online was acceptable since changes related to the COVID-19 pandemic, concerns were expressed regarding confidentiality when discussing sensitive issues online. There was a preference for in-person therapy, owing to the belief that this enhances therapist–patient communication and rapport. Misunderstanding that the intervention would be delivered by videocall was common but when participants realised it was mediated mostly via typing, they suggested this could be a barrier to conveying thoughts and emotions. Interview findings also indicated people self-excluded because they did not perceive themselves as depressed or because they viewed anxiety, or another mental health issue, as their primary mental health problem.

### Strengths and limitations

Quantitative data from 725 responders gave insight into the decision to decline, which was expanded on via 15 qualitative interviews. Sampling for interviews was based on four out of 12 response options, so not every reason for declining listed in the survey was explored in detail. However, sampling participants according to these four responses allowed us the greatest opportunity to explore patients’ views of the research and the intervention, which was the focus of the study. We acknowledge the differences in the quantitative survey results and qualitative interview findings. While the survey indicates that not wanting to participate in research was the most common reason for declining, the interview findings highlighted aspects of CBT that were disliked by participants. Those interviewed were purposefully sampled to ensure a range of views and perspectives, rather than to ensure a sample that was representative of those surveyed. Although the interview sample was small, it included people from a range of ages and genders, and resulted in a dataset that achieved information power.^18^ No information about the ethnicity of individuals who completed the reply slip and questionnaire was collected. Hence, the team could not purposefully sample to include the views of people from ethnic minoritised groups, who may be disproportionately affected by inequalities in access to mental health care.^22^

The data presented in this article were collected from individuals who had been approached about the study through a mailout from their GP practice. It is possible that if responders had been recruited by GPs during a routine consultation, some of the themes might not have been identified. For example, issues such as perceived ineligibility; feeling the timing of the request was not right; and believing that the intervention entailed mainly videocalls, could have been discussed with the recruiting GP at the time. In addition, misunderstanding about videocalls could be addressed by adding clarification to the study invitation letter and participant information leaflet.

### Comparison with existing literature

Previous research suggests that not wanting to take part in research is the most common reason for declining trial participation.^12,13,16^ The current study echoes this finding, highlighting a range of personal reasons, including the timing of the invitation, health concerns, and competing demands.

Depressive symptoms may adversely affect patients’ motivation to participate in research^14,15^ and they are likely to opt out if they judge themselves ineligible.^23^ The current findings indicate that people declined the trial invitation if they believed they were ineligible, which commonly related to the perception that they were not depressed. This belief was affected by psychiatric comorbidities; other more pressing mental health issues; changing depressive symptoms; or effective symptom management. The findings reiterate research that found patients’ ability and motivation to participate in research is also adversely affected if they have multiple mental health diagnoses or if they are extremely fragile.^14,15^

Non-participation in trials is commonly related to the intervention being evaluated.^12,13^ This study and previous research suggest prior negative experiences of the same (or similar) intervention influences non-participation.^17^ Current findings indicate that participants may dislike the active components of CBT such as completing worksheets and homework tasks. This is important because client tasks completed outside of therapy sessions are a core part of CBT and adherence to these can increase effectiveness.^24^ This learning underscores the need to personalise and adapt CBT tasks to individual patients in order to reduce any negative experiences and to explain to patients the value gained from engaging in CBT tasks.

Although delivering CBT online using instant messaging has been found to be clinically and cost-effective^7,25^, the current findings highlight concerns around typed communication, as well as mistrust about discussing sensitive issues online. Dislike of too much screen-based time was especially pertinent during the pandemic, although it was acknowledged that ‘remote’ or online medical consultations are becoming more commonplace and therefore an acceptable way to receive CBT. Recent evaluation concurs that while in-person contact may be preferable for mental health consultations, remote service delivery can be acceptable to patients.^26^ In addition, our recent qualitative work with INTERACT trial participants indicates that online CBT is acceptable and useful to some patients with depression.^27^

### Implications for research and practice

Recruitment of patients in general practice is key to generating evidence that will inform primary care policy and practice. However, trial recruitment in this setting remains a challenge.^28,29^ Many people declined to take part in the INTERACT trial because they did not want to receive the treatment offered. The nature of the intervention (specifically the mode of delivery via typing) was commonly misunderstood. This highlights the value of embedding work around decliners as part of an ongoing trial, to reduce ambiguity and ensure maximum clarity when describing the intervention in participant information materials. The current findings have implications both for trial teams and GPs in understanding patients’ preferences for treatments for depression and the likelihood that they will accept and engage with CBT delivered online.

The findings highlight that some reasons for declining may be ameliorated through GP discussions during ‘in consultation’ recruitment. Those invited may be averse to the type of therapy offered (CBT) or misunderstand the mode of delivery (online via typing). Others believe they are ineligible owing to their mental health status (that is, reduced depressive symptoms). Future research could consider tailoring the GP record search strategy, to identify people who had consulted more recently with depression. If individuals are approached about a study during a consultation (rather than via practice mailout) GPs can address patients’ concerns, such as their understanding of the acceptability of the trial intervention and their eligibility. Improving support for in-consultation recruitment should be a priority for the research team.^30^ The use of an electronic template, triggered at the point of consultation, has been shown to increase research recruitment in primary care, compared with searching for symptom codes and study mailouts. This effective prompt was also found to cause minimal disruption to the clinical consultation.^30^

Collective efforts between researchers and GPs could enhance trial recruitment within primary care, improving the evidence base for new ways of delivering interventions for people with depression.

## Trial registration number

ISRCTN13112900

## Data

Data from the INTERACT trial will be lodged with the University of Bristol Research Data Repository: https://data.bris.ac.uk/data and assigned a unique DOI. Access to INTERACT data for non-commercial research will be through a system of managed access (University of Bristol ‘controlled access’). The final decision to release data will be taken by the University of Bristol’s Data Access Committee. There may be a charge for preparing the dataset. Requests for data any other purposes must be negotiated separately.
